# ‘I don't know’—reclaiming not‐knowing in medical transitions

**DOI:** 10.1111/medu.15757

**Published:** 2025-06-13

**Authors:** Yvonne Carlsson, Matilda Liljedahl

**Affiliations:** ^1^ General Practice/Family Medicine, School of Public Health and Community Medicine, Institute of Medicine, Sahlgrenska Academy University of Gothenburg Gothenburg Sweden; ^2^ Department of Oncology, Institute of Clinical Sciences, Sahlgrenska Academy University of Gothenburg Gothenburg Sweden; ^3^ Department of Oncology Sahlgrenska University Hospital Gothenburg Sweden

## Abstract

The authors offer compelling argument for why admitting uncertainty should be considered a means of preparing for complex realities rather than treated as an indicator of incompetence.

Dineen and colleagues offer an insightful exploration of how newly qualified doctors experience and respond to uncertainty during their transition to internship.^1^ By centring the voices of interns and drawing on the integrative uncertainty tolerance (UT) model, they provide a nuanced account of challenges encountered during this transition—not only in delivering patient care but also in navigating unfamiliar environments, unclear roles and shifting professional identities. Their findings show that uncertainty is not only about clinical ambiguity, but deeply tied to role, context and relational dynamics.

Although uncertainty is often understood as a psychological or cognitive challenge, Dineen et al.'s findings suggest that it can also reflect deeper cultural expectations within the medical profession—such as knowing the etiquette and unwritten rules. We would like to build on this by drawing attention to how uncertainty may not only arise from what is unknown, but also from what is unspoken: the norms, ideals and ideologies shape what it means to know, to act and to be seen as competent. When a newly qualified doctor pauses before escalating care or hesitates to order a test, their internal dialogue is rarely just about uncertainty in terms of ‘How should I act?’. More often it is: ‘Can I act? Am I allowed to act? Do I know enough to act? How would I look if I acted?’. These are not simply individual reflections; they are shaped by team dynamics, workplace culture and expectations about what competent doctors do.

If uncertainty is shaped by unspoken norms and professional expectations, then we must also question the frameworks we use to study it. This brings us to the UT framework itself. It might offer a helpful way to describe how people experience uncertainty. But the framework stems from psychological traditions—especially cognitive and personality research—and focuses mostly on individual traits.^2^, ^3^ That makes us wonder: what might we miss when we frame uncertainty primarily as something to be ‘tolerated’? Does the language of tolerance and coping—though useful—subtly reinforce the idea that uncertainty is inherently negative, something to be endured and mitigated? For a reason, the field of health professions education has made a significant move towards acknowledging social aspects of learning that consider how uncertainty is shaped by culture, power, identity and the design of clinical work.^4^

Uncertainty is shaped by unspoken norms and professional expectations.

Taking a sociocultural perspective invites us to consider uncertainty not only as a problem to be managed, but as an integral part of the transition from student to doctor.^5^ From this view, while uncertainty might feel uncomfortable, it can also be an important signal that learning and development are underway. Instead of considering how to better prepare students and newly qualified doctors to tolerate uncertainty, we might ask: What role does uncertainty play in the process of becoming a doctor? When supported well, uncertain moments can be meaningful, even identity‐shaping.^6^ Rather than reflecting ‘poor preparation’, uncertain moments are part of how doctors learn to think, act and relate in new ways.

Rather than reflecting ‘poor preparation’, uncertain moments are part of how doctors learn.

A useful perspective on uncertainty is offered by the concept of *subjectification*.^7^ Subjectification highlights moments when individuals act not just in line with professional norms, but from their unique selves—as subjects who bring their judgement, identity and presence into clinical situations. Voicing uncertainty can be seen as one such act: a demonstration of responsibility where the individual acknowledges complexity, calls in others and openly reflects on their own limitations. From this view, saying ‘I don't know’ does not necessarily indicate incompetence—it signals that the learner is negotiating the tension between what the profession expects and their individual experience.

Saying ‘I don't know’ does not necessarily indicate incompetence.

It is a moment of becoming. Learning environments that support such expressions do not simply foster tolerance of uncertainty; it nurtures professionals who openly acknowledge complexity and act with integrity.

That would reposition uncertainty not as a failure but as an invitation to learn and to grow.

In their conclusion, Dineen et al. suggest reducing ‘non‐clinical uncertainties’—such as navigating electronic health record systems, referral pathways or finding equipment—through structured orientation programmes. As former interns, we recognise the sheer cognitive load involved in locating passwords, pagers, or even finding the right room. Yet it is precisely this kind of local knowledge—fluid, site‐specific and context‐bound—that resists standardisation. Designing pre‐internship orientation to cover every eventuality risks overpromising certainty in a system where variation is intrinsic. Rather than viewing such uncertainty as a barrier to be cleared before ‘real learning’ begins, we might better ask how learners can be supported to navigate uncertain situations.

What if we stopped viewing uncertainty as a problem to fix and instead recognised it as a normal and natural dimension of clinical work? What if we created cultures where people could say ‘I don't know’ without fear, and where uncertainty invited collaboration rather than shame? That would reposition uncertainty not as a failure but as an invitation to learn and to grow—and thus strengthen the relational fabric of medicine. Becoming a doctor, then, does not mean outgrowing uncertainty but learning how to authentically and confidently navigate it.

## AUTHOR CONTRIBUTIONS

**Yvonne Carlsson:** Conceptualization; methodology; writing—original draft; writing—review and editing. **Matilda Liljedahl:** Conceptualization; methodology; writing—review and editing.
